# Type VI Secretion System and Its Effectors PdpC, PdpD, and OpiA Contribute to *Francisella* Virulence in Galleria mellonella Larvae

**DOI:** 10.1128/IAI.00579-20

**Published:** 2021-06-16

**Authors:** Maj Brodmann, Sophie T. Schnider, Marek Basler

**Affiliations:** a Biozentrum, University of Basel, Basel, Switzerland; Stanford University

**Keywords:** *Francisella tularensis* subsp. *novicida*, *Galleria mellonella*, tularemia, *in vivo* infection model, type VI secretion system activation and effectors, polyethylene glycol, T6SS

## Abstract

Francisella tularensis causes the deadly zoonotic disease tularemia in humans and is able to infect a broad range of organisms including arthropods, which are thought to play a major role in *Francisella* transmission. However, while mammalian *in vitro* and *in vivo* infection models are widely used to investigate *Francisella* pathogenicity, a detailed characterization of the major *Francisella* virulence factor, a noncanonical type VI secretion system (T6SS), in an arthropod *in vivo* infection model is missing. Here, we use Galleria mellonella larvae to analyze the role of the *Francisella* T6SS and its corresponding effectors in F. tularensis subsp. *novicida* virulence. We report that G. mellonella larvae killing depends on the functional T6SS and infectious dose. In contrast to other mammalian *in vivo* infection models, even one of the T6SS effectors PdpC, PdpD, or OpiA is sufficient to kill G. mellonella larvae, while sheath recycling by ClpB is dispensable. We further demonstrate that treatment by polyethylene glycol (PEG) activates *Francisella* T6SS in liquid culture and that this is independent of the response regulator PmrA. PEG-activated IglC secretion is dependent on T6SS structural component PdpB but independent of putative effectors PdpC, PdpD, AnmK, OpiB_1_, OpiB_2_, and OpiB_3_. The results of larvae infection and secretion assay suggest that AnmK, a putative T6SS component with unknown function, interferes with OpiA-mediated toxicity but not with general T6SS activity. We establish that the easy-to-use G. mellonella larvae infection model provides new insights into the function of T6SS and pathogenesis of *Francisella*.

## INTRODUCTION

Francisella tularensis is the causative agent of the deadly zoonotic disease called tularemia (1). The most virulent subspecies Francisella tularensis subsp. *tularensis* is considered a tier 1 select agent due to high infectivity in humans (50% lethal dose, <10 CFU) and a high mortality rate if left untreated (up to 60%) (1, 2). In Europe, the less virulent Francisella tularensis subsp. *holarctica* is most prevalent (1). The closely related Francisella tularensis subsp. *novicida* is often used as a model organism to study *Francisella* pathogenicity, as it has a high infectivity in mice but not in humans (2).

*Francisella* virulence depends on the *Francisella* pathogenicity island (FPI) (3). Interestingly, F. tularensis subsp. *novicida* encodes one FPI, while the more virulent subspecies F. tularensis subsp. *tularensis* and F. tularensis subsp. *holarctica* both possess two identical FPIs (4). The FPI encodes a noncanonical type VI secretion system (T6SS) (see Fig. S1 in the supplemental material) required for intracellular survival (4–7). T6SS is a contractile nanomachine that can translocate effector proteins into bacterial and eukaryotic cells (8). The T6SS consists of the following three subcomplexes: a membrane complex spanning the bacterial cell envelope; a baseplate complex harboring the spike and effectors; and a contractile, cytosolic sheath with inner tube (8). Contraction of the cytosolic sheath propels the inner tube with the spike and effectors into a target cell (9–11). Dynamics of sheath assembly and contraction can be visualized by live-cell fluorescence microscopy and serves as a readout for a functional T6SS (5, 12, 13).

For F. tularensis subsp. *novicida*, six secreted T6SS effectors were identified as follows: PdpC, PdpD, OpiA, and OpiB_1_, OpiB_2_, and OpiB_3_ (OpiB_1–3_) (14). While PdpC and PdpD are required for phagosomal escape, their exact mode of action remains elusive (15, 16). Interestingly, OpiA and OpiB_1–3_ are encoded outside the FPI at different genomic sites (14). OpiA was shown to be a bacterial phosphatidylinositol 3-kinase delaying phagosomal maturation (17). Conversely, the function of the three almost identical OpiB proteins is unclear (14). The FPI encodes additional components PdpE and AnmK, which are dispensable for T6SS assembly and dynamics, and thus may be putative effectors (5). However, the corresponding deletion mutants were indistinguishable from the parental strains in various infection models (5, 15, 18, 19).

Strikingly, *Francisella* is able to infect and survive in a wide range of hosts ranging from amoeba and insects to mammals (20–24). Although the primary niche of *Francisella* is phagocytic cells, such as macrophages, *Francisella* is able to infect a broad range of cells, including nonphagocytic cells such as HeLa cells, Drosophila melanogaster cells, or erythrocytes (6, 25–28). Furthermore, there is clear evidence that tularemia is transmitted either by aerosols, infected animals, or by arthropod vectors such as ticks (21, 29, 30). Particularly, the broad range of arthropods that are susceptible for *Francisella* infections suggests that arthropods may play a role in maintaining *Francisella* in the environment (24).

An increasingly used arthropod *in vivo* infection model for studying host-pathogen interactions as well as for antimicrobial drug testing is Galleria mellonella larva (31). G. mellonella larvae combine several advantages for research, such as low maintenance costs and few ethical problems, compared to mammalian *in vivo* infection models (32). Moreover, G. mellonella larvae contain a complex innate immune system, including phagocytic cells called hemocytes and a humoral response (31). A part of the humoral response is a melanization process required for encapsulation of pathogens (33). Melanization results in a color change of the larvae from a healthy yellow into different shades of brown and black depending on the strength of the immune response (34). Recently, the complete G. mellonella genome was sequenced, facilitating genetic manipulations in the future (35).

G. mellonella larvae were already used as an *in vivo* infection model for *Francisella*. However, these studies focused on initial characterization of inoculum concentrations and infection conditions for robust killing of G. mellonella larvae by various *Francisella* species (36–39). Crucially, in-depth characterization of the major *Francisella* virulence factor, the noncanonical *Francisella* T6SS, and its role in killing of G. mellonella larvae is lacking.

Here, we show that virulence of F. tularensis subsp. *novicida* in G. mellonella larvae depends on a functional T6SS. However, ClpB-mediated T6SS sheath recycling is less important than reported previously in mice and bone marrow-derived macrophages (BMDMs). In addition, the main T6SS effectors PdpC and PdpD were dispensable for killing G. mellonella larvae. In contrast to mammalian *in vivo* infection models, individual effectors PdpC, PdpD, or OpiA were sufficient to kill G. mellonella larvae in a manner comparable to the parental strain. We demonstrate that *Francisella* T6SS can be activated *in vitro* by polyethylene glycol (PEG) in a PmrA-independent manner and use this to show that AnmK affects OpiA-mediated killing of G. mellonella larvae without altering T6SS activity or IglC secretion. In summary, our results suggest that G. mellonella larvae serve as a suitable model for testing roles of uncharacterized *Francisella* genes in infection.

## RESULTS

### T6SS is required for efficient killing of larvae by F. tularensis subsp. *novicida.*

**FIG 1:**
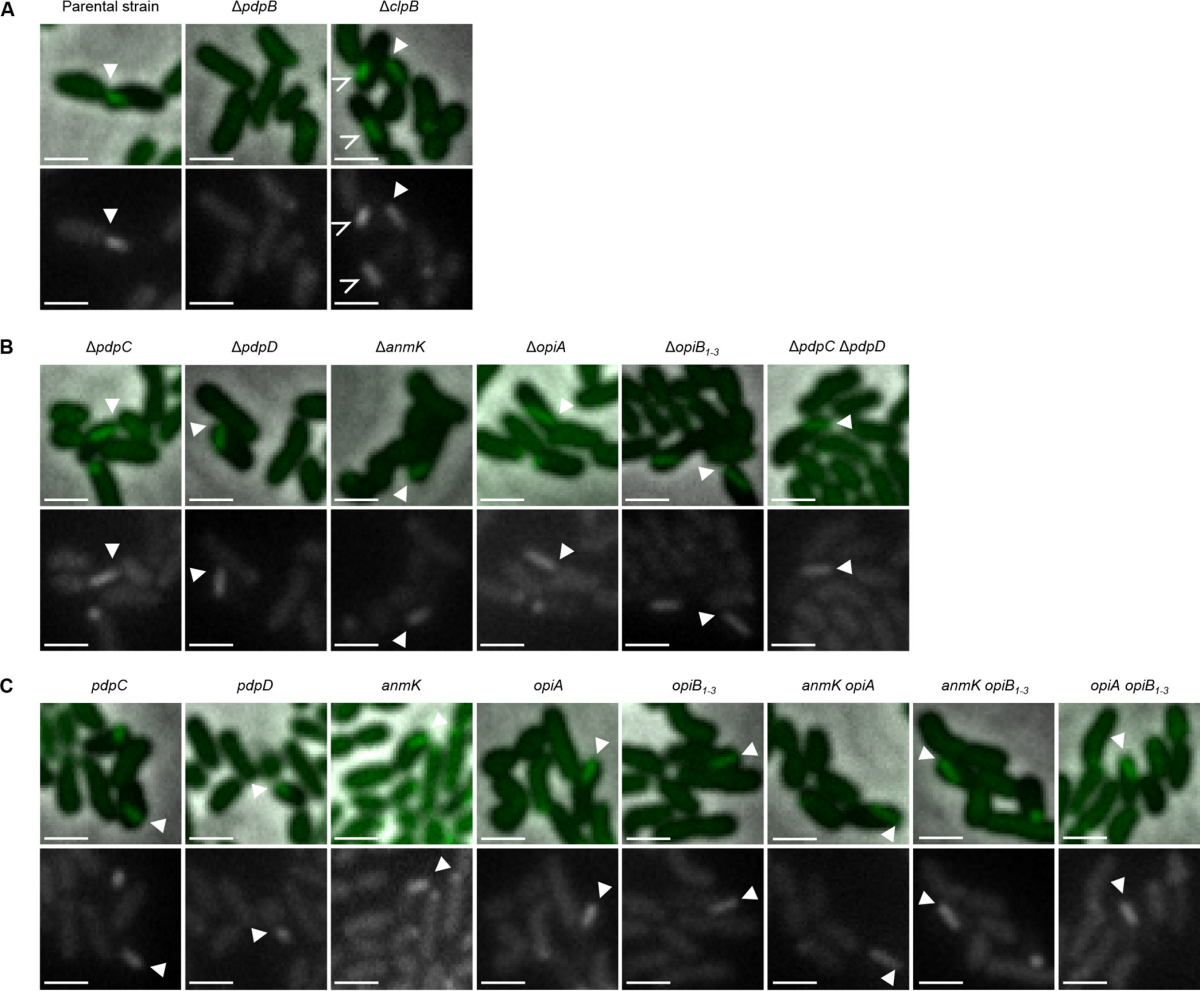
T6SS assembly in F. tularensis subsp. *novicida* is independent of PdpC, PdpD, AnmK, OpiA, and OpiB_1–3_. All F. tularensis subsp. *novicida* mutants used in this study exhibit a functional T6SS. Filled arrows point to examples of assembled T6SS. Upper images are a merge of phase contrast and GFP channel. The lower images show GFP channel only. The 3.3- by 3.3-μm fields of view are shown. Scale bars represent 1 μm. (A) Assembly of IglA-sfGFP containing T6SS sheath in F. tularensis subsp. *novicida* U112 *iglA-sfGFP* (parental strain) and the Δ*clpB* mutant. Empty arrows point to sfGFP aggregates in the F. tularensis subsp. *novicida* U112 *iglA-sfGFP* Δ*clpB* strain. No T6SS assembly was observed in the F. tularensis subsp. *novicida* U112 *iglA-sfGFP* Δ*pdpB* strain (T6SS-negative control). (B) Assembly of IglA-sfGFP containing T6SS sheath in F. tularensis subsp. *novicida* U112 *iglA-sfGFP* Δ*pdpC*, Δ*pdpD*, Δ*anmK*, Δ*opiA*, Δ*opiB_1_*_–_*_3_*, and Δ*pdpC* Δ*pdpD* strains. (C) Assembly of IglA-sfGFP containing T6SS sheath in F. tularensis subsp. *novicida* U112 *iglA-sfGFP* Δ*pdpD* Δ*anmK* Δ*opiA* Δ*opiB_1_*_–_*_3_* (*pdpC*), Δ*pdpC* Δ*anmK* Δ*opiA* Δ*opiB_1_*_–_*_3_* (*pdpD*), Δ*pdpC* Δ*pdpD* Δ*opiA* Δ*opiB_1_*_–_*_3_* (*anmK*), Δ*pdpC* Δ*pdpD* Δ*anmK* Δ*opiB_1_*_–_*_3_* (*opiA*), Δ*pdpC* Δ*pdpD* Δ*anmK* Δ*opiA* (*opiB_1_*_–_*_3_*), Δ*pdpC* Δ*pdpD* Δ*opiB_1_*_–_*_3_* (*anmK opiA*), Δ*pdpC* Δ*pdpD* Δ*opiA* (*anmK opiB_1_*_–_*_3_*), and Δ*pdpC* Δ*pdpD* Δ*anmK* (*opiA opiB_1_*_–_*_3_*) strains.

**FIG 2:**
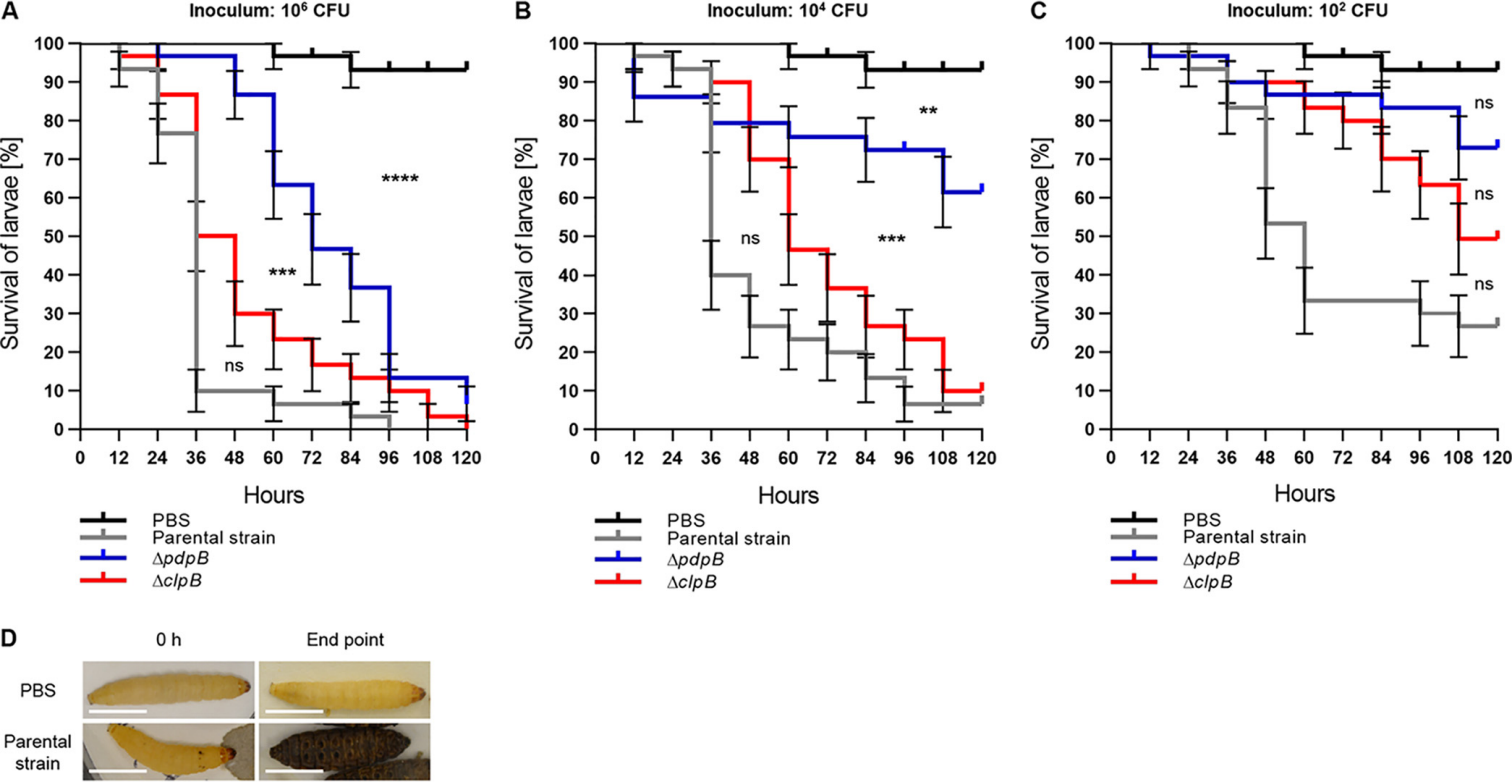
F. tularensis subsp. *novicida* kills G. mellonella larvae in a T6SS- and concentration-dependent manner. (A to C) Survival curves represent three individual experiments over 5 days pooled together (*n*_0 total_ = 30; *n*_0_ = 10 per experiment). State of G. mellonella larvae was monitored every 12 h. Pupating larvae were censored (vertical dashes). Error bars indicate standard error. Black survival curves, PBS-treated G. mellonella larvae; gray survival curves, G. mellonella infected with F. tularensis subsp. *novicida* U112 *iglA-sfGFP* (parental strain); blue survival curves, G. mellonella *larvae* infected with F. tularensis subsp. *novicida* U112 *iglA-sfGFP* Δ*pdpB* strain (T6SS-negative control); red survival curves, G. mellonella infected with F. tularensis subsp. *novicida* U112 *iglA-sfGFP* Δ*clpB* strain. Individual curves were compared with log rank (Mantel-Cox) test. *P* values above a Bonferroni-corrected threshold were considered nonsignificant (ns). **, *P* < 0.01; ***, *P *< 0.001; ****, *P *< 0.0001. Following curves were compared. Parental strain versus Δ*clpB* mutant, Δ*clpB* mutant versus Δ*pdpB* mutant, and Δ*pdpB* mutant versus PBS control. Calculated infection inocula are as follows: 10^6^ CFU (A), 10^4^ CFU (B), and 10^2^ CFU (C). (D) Representative examples of G. mellonella larvae morphology directly after PBS treatment and infection with F. tularensis subsp. *novicida* U112 *iglA-sfGFP* (parental strain) at an infection dose of 10^4^ CFU and after 120 h or 96 h, respectively.

In order to characterize G. mellonella larvae as an *in vivo* infection model for *Francisella*, we first tested if F. tularensis subsp. *novicida* establishes infection in a T6SS-dependent manner. We used F. tularensis subsp. *novicida* U112 *iglA-sfGFP* (10) as the parental strain, which has T6SS sheath component IglA labeled with superfolder green fluorescent protein (sfGFP) and, thus, allows monitoring of T6SS dynamics by live-cell fluorescence microscopy (Fig. 1A; see also Movies S1 and S2 in the supplemental material) (5). We compared survival of G. mellonella larvae infected with the parental strain and that of a T6SS-negative control, in which *pdpB*, part of the T6SS membrane complex, was deleted (Fig. 1A; see also Fig. S1 and Movies S1 and S2 in the supplemental material) (5). G. mellonella survival was monitored for three different calculated inocula (10^6^ CFU, 10^4^ CFU, and 10^2^ CFU per injection) and compared to a phosphate-buffered saline (PBS) control (Fig. 2A to C). Fifty percent of larvae infected with the parental strain were dead after 36 to 60 h, depending on the infection dose, while significantly more larvae infected with the T6SS-negative strain remained alive (Fig. 2A to C). PBS-treated G. mellonella larvae survived over 120 h (Fig. 2A to C). The infection dose of 10^4^ CFU killed 93% of larvae when infected with the parental strain; however, less than 40% of larvae died when infected with the T6SS-negative strain after 120 h (Fig. 2B). Moreover, the parental F. tularensis subsp. *novicida* strain was able to robustly trigger an immune response in G. mellonella larvae, indicated by the melanization process and darkening of the larvae (Fig. 2D). In general, the killing rate for each strain and infection dose was reproducible over three independent infection experiments (see Fig. S2 in the supplemental material). While our data show that the *Francisella* T6SS is a major virulence factor in G. mellonella larvae, at higher infection doses, larvae infected with the T6SS-negative strain were also killed, suggesting that additional virulence factors play a role during infection.

### ClpB and effectors PdpD and PdpC are less important for establishing infection in G. mellonella larvae than in mammalian infection models.

ClpB-mediated refolding of the T6SS sheath is essential for *Francisella* virulence in BMDMs and mice (5, 40). To test the role of ClpB in G. mellonella, we infected the larvae with a Δ*clpB* mutant (Fig. 1A; see also Fig. S1 and Movies S1 and S2). Surprisingly, a Δ*clpB* mutant killed G. mellonella larvae almost as efficiently as the parental strain (Fig. 2A to C). An average delay in killing of approximately 12 to 24 h was observed for the Δ*clpB* mutant for all infection doses, suggesting that while ClpB contributes to infection, it is largely dispensable.

**FIG 3:**
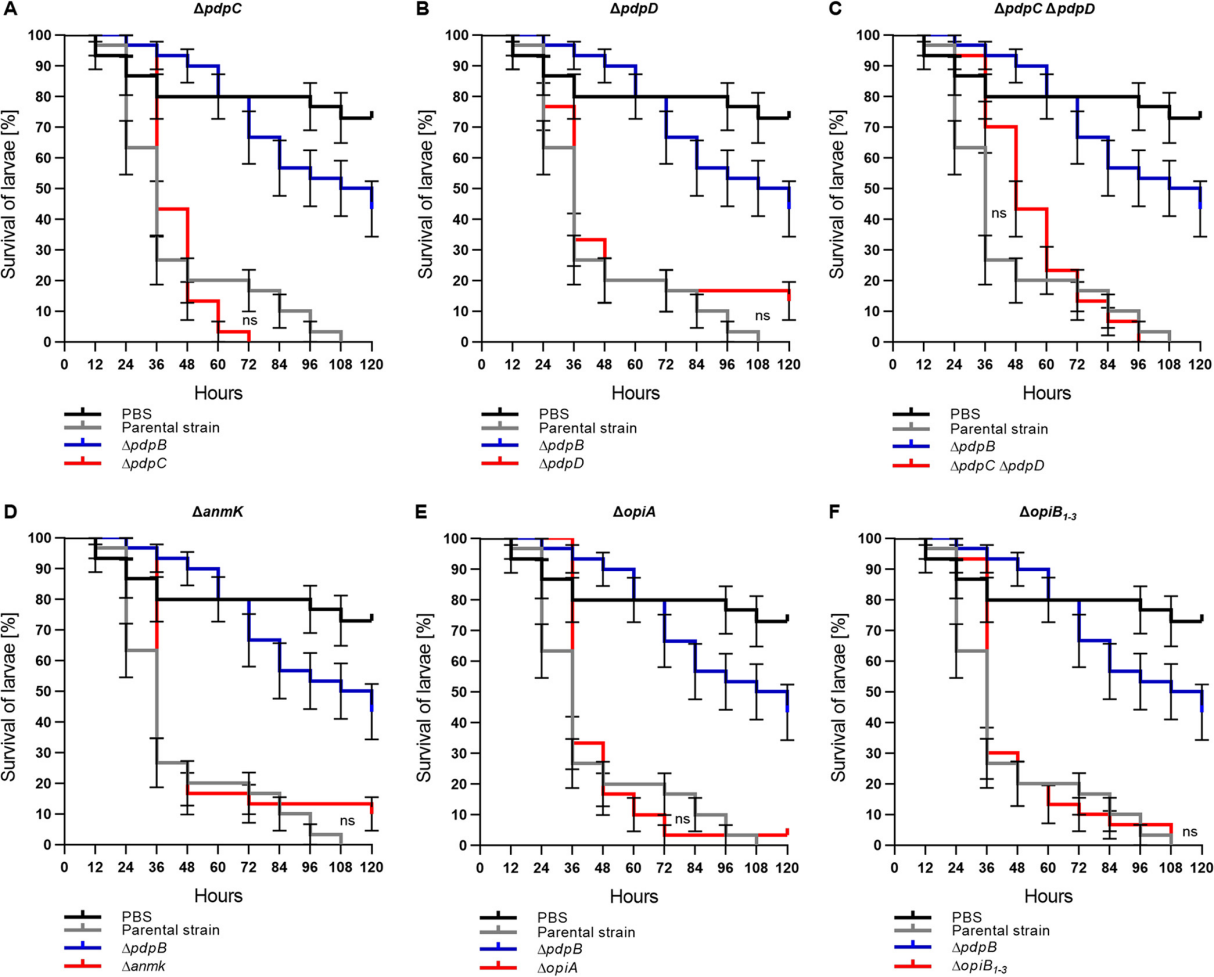
PdpC and PdpD are dispensable for T6SS-dependent *Francisella* virulence in G. mellonella. Survival curves represent three individual experiments over 5 days pooled together (*n*_0 total_ = 30; *n*_0_ = 10 per experiment). State of G. mellonella larvae was monitored every 12 h. Pupating larvae were censored (vertical dashes). Error bars indicate standard error. Individual curves were compared with log rank (Mantel-Cox) test. *P* values above a Bonferroni-corrected threshold were considered nonsignificant (ns). Parental strain versus mutant curves were compared. Black survival curves, PBS-treated G. mellonella *larvae*; gray survival curves, G. mellonella infected with F. tularensis subsp. *novicida* U112 *iglA-sfGFP* (parental strain); blue survival curves, G. mellonella larvae infected with F. tularensis subsp. *novicida* U112 *iglA-sfGFP* Δ*pdpB* strain (T6SS-negative control); red survival curves, G. mellonella infected with F. tularensis subsp. *novicida* U112 *iglA-sfGFP* (A) Δ*pdpC* mutant; (B) Δ*pdpD* mutant; (C) Δ*pdpC* Δ*pdpD* mutant; (D) Δ*anmK* mutant; (E) Δ*opiA* mutant; (F) Δ*opiB_1_*_–_*_3_* mutant.

Next, we focused on the role of the FPI components, which are not required for T6SS assembly (5) (Fig. 1B; see also Fig. S1 and Movies S1 and S2). First, we tested the role of T6SS effectors PdpC and PdpD, which are secreted in a T6SS-dependent manner and have a major role in phagosomal escape in BMDMs and mice (5, 14–16). Since the infection with 10^4^ CFU (Fig. 2B) resulted in the biggest survival difference between the parental strain and a T6SS-negative strain, we used this dose for all remaining infections. Surprisingly, single in-frame deletions of *pdpC* and *pdpD* had no effect on *Francisella* virulence in larvae (Fig. 3A and B). Even a Δ*pdpC* Δ*pdpD* double mutant, which is avirulent in BMDM and mice (5), killed the G. mellonella larvae as efficiently as the parental strain (Fig. 3C). We further analyzed the contribution of AnmK, a FPI component of unknown function (4) and the four secreted effectors OpiA and OpiB_1–3_, which are located outside of the FPI (14, 17) (Fig. 1B; see also Fig. S1 and Movies S1 and S2). Single deletion of either of the genes encoding these proteins had no effect on *Francisella* virulence in larvae (Fig. 3D to F; see also Fig. S3 in the supplemental material), suggesting that T6SS effectors play redundant roles in killing of G. mellonella.

### PdpC, PdpD, and OpiA alone are sufficient for killing of larvae.

**FIG 4:**
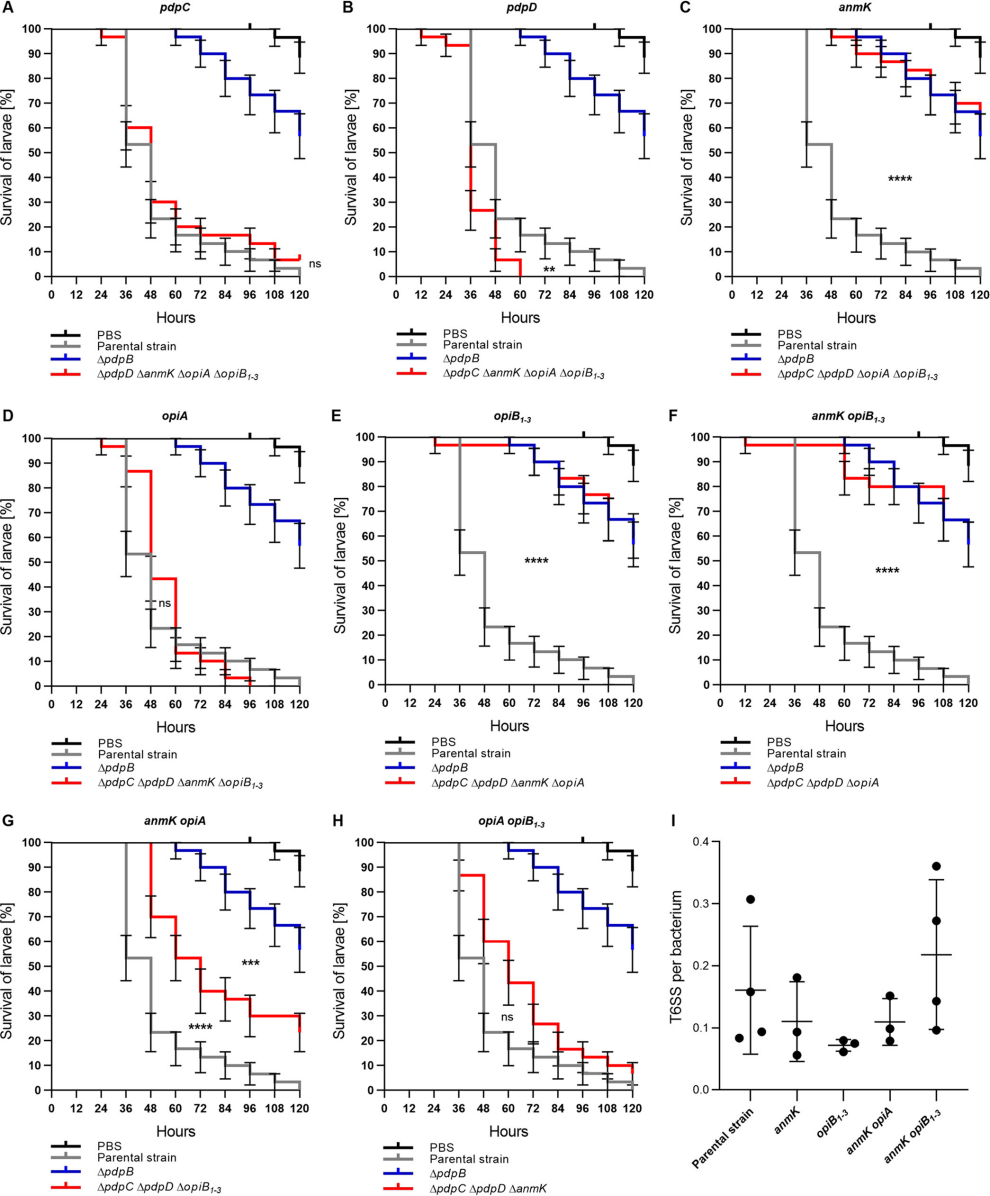
PdpC, PdpD, and OpiA are sufficient for T6SS-dependent *Francisella* virulence in G. mellonella. Survival curves represent three individual experiments over 5 days pooled together (*n*_0 total_ = 30; *n*_0_ = 10 per experiment). State of G. mellonella larvae was monitored every 12 h. Pupating larvae were censored (vertical dashes). Error bars indicate standard error. Individual curves were compared with log rank (Mantel-Cox) test. *P* values above a Bonferroni-corrected threshold were considered nonsignificant (ns). **, *P* < 0.01; ***, *P* < 0.001; ****, *P *< 0.0001. Parental strain versus mutant curves were compared to each other. For panel G, mutant versus Δ*pdpB* strain curve was also compared. Black survival curves, PBS-treated G. mellonella larvae; gray survival curves, G. mellonella infected with F. tularensis subsp. *novicida* U112 *iglA-sfGFP* (parental strain); blue survival curves, G. mellonella *larvae* infected with F. tularensis subsp. *novicida* U112 *iglA-sfGFP* Δ*pdpB* strain (T6SS-negative control); red survival curves, G. mellonella infected with F. tularensis subsp. *novicida* U112 *iglA-sfGFP* Δ*pdpD* Δ*anmK* Δ*opiA* Δ*opiB_1_*_–_*_3_* mutant (*pdpC*) (A), Δ*pdpC* Δ*anmK* Δ*opiA* Δ*opiB_1_*_–_*_3_* mutant (*pdpD*) (B), Δ*pdpC* Δ*pdpD* Δ*opiA* Δ*opiB_1_*_–_*_3_* mutant (*anmK*) (C), Δ*pdpC* Δ*pdpD* Δ*anmK* Δ*opiB_1_*_–_*_3_* mutant (*opiA*) (D), Δ*pdpC* Δ*pdpD* Δ*anmK* Δ*opiA* mutant (*opiB_1_*_–_*_3_*) (E), Δ*pdpC* Δ*pdpD* Δ*opiA* mutant (*anmK opiB_1_*_–_*_3_*) (F), Δ*pdpC* Δ*pdpD* Δ*opiB_1_*_–_*_3_* mutant (*anmK opiA*) (G), and Δ*pdpC* Δ*pdpD* Δ*anmK* mutant (*opiA opiB_1_*_–_*_3_*) (H). (I) Quantification of T6SS sheaths per bacterium within 5 min for F. tularensis subsp. *novicida* U112 *iglA-sfGFP* (parental strain), Δ*pdpC* Δ*pdpD* Δ*opiA* Δ*opiB_1_*_–_*_3_* mutant (*anmK*), Δ*pdpC* Δ*pdpD* Δ*anmK* Δ*opiA* mutant (*opiB_1_*_–_*_3_*), Δ*pdpC* Δ*pdpD* Δ*opiB_1_*_–_*_3_* mutant (*anmK opiA*), and Δ*pdpC* Δ*pdpD* Δ*opiA* mutant (*anmK opiB_1_*_–_*_3_*). At least three biological replicates with at least 3,200 bacteria each were analyzed per strain. Mean with standard deviation is shown. No significant differences in means were detected with Tukey’s multiple-comparison test and 95% confidence level.

Since both PdpC and PdpD are dispensable for *Francisella* virulence in G. mellonella larvae, we hypothesized that either other effectors, such as OpiA and OpiB_1–3_, may compensate for the loss of PdpC and PdpD or that *Francisella* secretes additional T6SS effectors. To distinguish between these two possibilities, we first assessed if the previously identified T6SS effectors are individually sufficient to kill G. mellonella. We prepared strains where we deleted genes encoding all but one of the known or suspected effectors (PdpC, PdpD, AnmK, OpiA, and OpiB_1–3_) (see Fig. S1). Interestingly, the strains with PdpC, PdpD, and OpiA alone were as efficient in killing of larvae as the parental strain (Fig. 4A, B, and D; see also Fig. S4 in the supplemental material). A strain expressing only PdpD killed larvae even faster than the parental strain (Fig. 4B). In contrast, F. tularensis subsp. *novicida* strains with *pdpC*, *pdpD*, and *opiA* deleted (only *anmK* and/or *opiB_1_*_–_*_3_* present) killed larvae at the same rate as the T6SS-negative strain (Δ*pdpB* mutant) (Fig. 4C, E, and F; see also Fig. S4). Interestingly, killing of G. mellonella larvae was significantly delayed in a strain with both *anmK* and *opiA* present compared to a strain having only *opiA* (Fig. 4G; see also Fig. S4). In contrast, the presence of *opiB_1_*_–_*_3_* had no significant effect on OpiA-mediated killing of larvae (Fig. 4H; see also Fig. S4). Importantly, all of these strains assembled T6SS with a frequency and dynamics comparable to those of the parental strain (Fig. 1C and Fig. 4I; see also Movie S1 and S2). In summary, these results suggest that PdpC, PdpD, or OpiA are individually sufficient to kill G. mellonella larvae and that AnmK specifically reduces OpiA-mediated killing.

### Polyethylene glycol activates *Francisella* T6SS assembly and IglC secretion.

**FIG 5:**
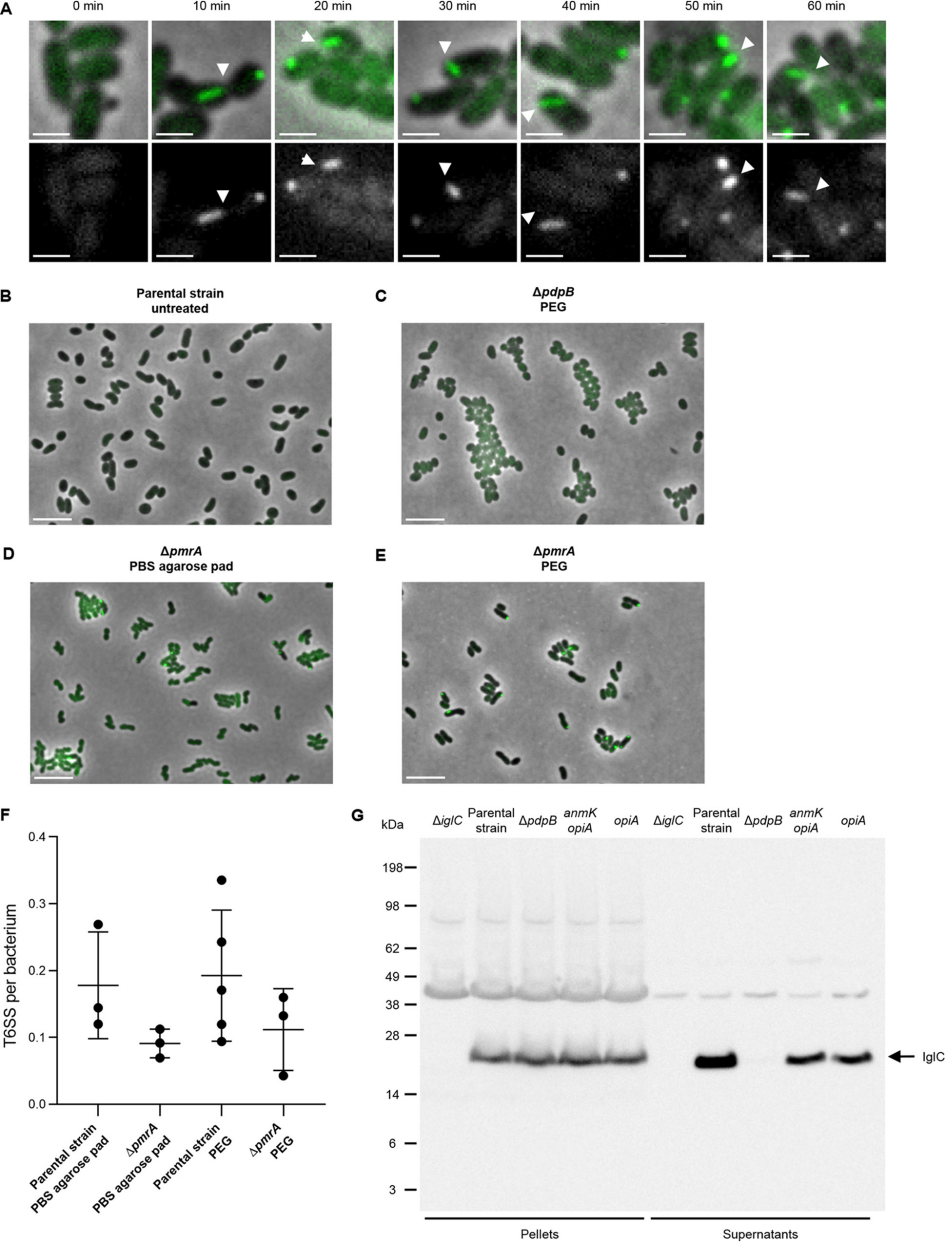
PEG activates *Francisella* T6SS in liquid culture. (A) Examples of assembled T6SS (IglA-sfGFP) in F. tularensis subsp. *novicida* U112 *iglA-sfGFP* (parental strain) during PEG treatment. Filled arrows point to examples of assembled T6SS. Upper images are a merge of phase contrast and GFP channel. The lower images show GFP channel only. The 3.3- by 3.3-μm fields of views are shown. Scale bars represent 1 μm. (B) No T6SS assemblies (IglA-sfGFP) were observed in untreated F. tularensis subsp. *novicida* U112 *iglA-sfGFP* (parental strain) and in the Δ*pdpB* mutant (T6SS-negative control) (C) after PEG treatment for 60 min. (D) T6SS activation in F. tularensis subsp. *novicida* U112 *iglA-sfGFP* Δ*pmrA* mutant on PBS agarose pad after 60 min incubation. (E) T6SS assemblies in F. tularensis subsp. *novicida* U112 *iglA-sfGFP* Δ*pmrA* mutant after PEG treatment for 60 min. (B to E) Merge of phase contrast and GFP channel and 39- by 26-μm fields of view are shown. Scale bars represent 5 μm. (F) Quantification of T6SS sheaths per bacterium within 5 min. At least three biological replicates with at least 750 bacteria each were analyzed per strain and condition. Mean with standard deviation is shown. No significant differences in means were detected with Tukey’s multiple-comparison test and 95% confidence level. (G) Levels of inner tube protein IglC was assessed in bacterial pellets and concentrated supernatants of F. tularensis subsp. *novicida* U112 *iglA-sfGFP* (parental strain), Δ*iglC* mutant (negative control for α-IglC antibody), Δ*pdpB* mutant (T6SS-negative control), Δ*pdpC* Δ*pdpD* Δ*opiB_1_*_–_*_3_* mutant (*anmK opiA*), and Δ*pdpC* Δ*pdpD* Δ*anmK* Δ*opiB_1_*_–_*_3_* mutant (*opiA*) after PEG treatment for 1 h. Arrow points to IglC bands (theoretical size, 22.1 kDa). An exposure time of 1 min was used for developing the immunoblot.

Previous work identified activation of T6SS expression and assembly by 5% KCl treatment (10) or by 30 to 60 min of incubation on PBS-agarose pads (5). Inspired by the observation that T6SS in Vibrio fischeri is activated by increasing the viscosity of the medium (41), we tested if similar conditions could activate F. tularensis subsp. *novicida* T6SS. We show that treatment of an exponentially growing culture of F. tularensis subsp. *novicida* U112 *iglA-sfGFP* by 10% polyethylene glycol 4000 (PEG) induces assembly of IglA-sfGFP into dynamic structures in less than 20 min (Fig. 5A). In contrast, no such structures were detected in untreated cells (Fig. 5B) or PEG-treated T6SS-negative mutant (Δ*pdpB* mutant) cells (Fig. 5C).

In *Francisella*, the orphan response regulator PmrA is required for regulation of FPI expression and intracellular replication upon environmental cues (42–44). Thus, we hypothesized that PmrA could be involved in agarose pad-dependent and/or PEG-dependent *Francisella* T6SS activation. However, deletion of *pmrA* did not significantly change T6SS activity compared to the parental strain with either of the two activation methods (Fig. 5D to F). Moreover, we observed that T6SS dynamics and number of T6SS assemblies per cell upon PEG treatment were comparable to what was observed upon starvation on PBS-agarose pads (Fig. 5F).

The advantage of PEG treatment is that it activates T6SS assembly in liquid culture similarly to the previously used 5% KCl treatment (10, 14). Therefore, we tested if PEG treatment also results in T6SS-dependent IglC secretion. Indeed, IglC was secreted by T6SS-positive parental strain while no IglC was detected in the supernatant of the T6SS-negative mutant (Δ*pdpB* mutant) (Fig. 5G). We also used this IglC secretion assay to rule out that the observed AnmK-dependent modulation of F. tularensis subsp. *novicida* infection is due to its role in T6SS-mediated secretion. Importantly, both strains containing only *opiA* or *opiA* and *anmK* secreted IglC at comparable levels, albeit at a slightly lower level than that of the parental strain (Fig. 5G). This suggests that a general defect in T6SS function is an unlikely explanation for the observed AnmK-dependent decrease in virulence toward G. mellonella (Fig. 4G).

## DISCUSSION

Since *Francisella* is able to infect a broad range of arthropods, it is important to understand *Francisella* pathogenicity in suitable infection models. Here, we characterized the contribution of the noncanonical T6SS and its known effectors to F. tularensis subsp. *novicida* pathogenicity in an *in vivo* arthropod infection model, namely, G. mellonella larvae. Our data show that F. tularensis subsp. *novicida* robustly kills G. mellonella larvae in a T6SS- and dose-dependent manner (Fig. 2; see also Fig. S2 in the supplemental material). These findings are in agreement with reports for other established *in vivo* infection models (4, 5, 45). Moreover, we could replicate the different dose-dependent killing dynamics with the parental strain reported previously (39). Interestingly, even a T6SS-negative strain killed some G. mellonella larvae, especially at high infection doses (Fig. 2; see also Fig. S2), suggesting that other virulence factors contribute to *Francisella* virulence in G. mellonella larvae. Indeed, *Francisella* encodes a variety of other bacterial virulence factors, such as type II secretion systems, type IV pili, outer membrane vesicles, nutritional virulence factors, as well as mechanisms to avoid oxidative stress and immune recognition (46–54).

We observed striking differences in the importance of individual T6SS components for F. tularensis subsp. *novicida* virulence in G. mellonella larvae compared to other mammalian *in vivo* infection models such as mice. First, T6SS sheath recycling and thus repeated T6SS firing is less important for *Francisella* pathogenicity in G. mellonella larvae than in BMDMs and mice (5). A Δ*clpB* mutant killed G. mellonella larvae slower but to the same extent as the parental strain (Fig. 2; see also Fig. S2). In contrast, a Δ*clpB* mutant was attenuated in Drosophila melanogaster, another arthropod *in vivo* infection model (25). It is important to note that the Δ*clpB* mutant is likely able to secrete a limited number of effectors because assembly and contraction of the T6SS sheath is independent of ClpB (5). Thus, one explanation for the observed difference between G. mellonella larvae and other *in vivo* infection models could be that G. mellonella cells are more sensitive to T6SS effectors or less capable of inhibiting the bacteria, and thus less effector translocation is sufficient for *Francisella* survival. Indeed, F. tularensis subsp. *tularensis* and F. tularensis subsp. *holarctica* Δ*clpB* mutants are reported to replicate to higher numbers in J774A.1 cells than in bone marrow-derived macrophages, suggesting that some cell types may be more sensitive to T6SS effectors than others (40). Interestingly, F. tularensis subsp. *tularensis* and F. tularensis subsp. *holarctica* Δ*clpB* mutants were less attenuated in mice than the F. tularensis subsp. *novicida* Δ*clpB* mutant (5, 40). However, both F. tularensis subsp. *tularensis* and F. tularensis subsp. *holarctica* encode two T6SS (4) and thus are potentially capable of secreting more effectors even with impaired T6SS compared to F. tularensis subsp. *novicida*. In summary, the general sensitivity to T6SS effectors as well as the number of translocation events may at least partially explain the variety of *Francisella* Δ*clpB* mutant phenotypes in different infection models.

Another striking difference in G. mellonella larvae compared to mice and other mammalian infection models is that *Francisella* virulence did not solely depend on T6SS effectors PdpC and PdpD (Fig. 3A to C; see also Fig. S3 in the supplemental material) (4, 5, 15, 16, 29, 55, 56). These results suggest that *Francisella* may manipulate different host cell components in insects and in mammal infection models or that arthropods are more sensitive to other T6SS effectors, such as OpiA. In agreement, single interruptions of *pdpC* and *pdpD* by transposons had no effect on *Francisella* virulence in Drosophila melanogaster or in a cell line derived from Anopheles gambiae (25, 57).

Interestingly, individual PdpC, PdpD, or OpiA effectors were sufficient to mediate *Francisella* virulence in G. mellonella larvae (Fig. 4A, B, and D; see also Fig. S4 in the supplemental material), which explains why deletion of *pdpC* and *pdpD* results in no change in virulence (Fig. 3C). In agreement, redundant functions for PdpC and OpiA were previously proposed (17). A strain with *pdpC*, *anmK*, *opiA*, and *opiB_1_*_–_*_3_* deleted and only left with *pdpD* was even significantly faster in killing G. mellonella larvae than the parental strain (Fig. 4B). It remains to be determined if this is due to increased translocation rate of PdpD in the absence of other effectors.

While we cannot rule out that F. tularensis subsp. *novicida* encodes additional yet unidentified T6SS effectors, deletion of *pdpC*, *pdpD*, and *opiA* resulted in an attenuated phenotype in G. mellonella larvae comparable to that of a T6SS-negative mutant (Fig. 4F), and mutants with only *anmK* or *opiB_1_*_–_*_3_* were also severely attenuated (Fig. 4C and E). Therefore, we conclude that PdpC, PdpD, and OpiA are the most important effectors for *Francisella* virulence in G. mellonella larvae.

Surprisingly, OpiA-mediated toxicity was affected by AnmK while general T6SS-dependent secretion was comparable to that of a fully virulent single *opiA* mutant (Fig. 4G and 5G; see also Fig. S4). Previously, no function of AnmK was observed in mice or macrophages (5, 15, 19, 25, 58). In contrast to OpiA, AnmK was never shown to be secreted (14). AnmK is predicted to contain an anhydro-*N*-acetylmuramic acid kinase domain, which is normally involved in peptidoglycan recycling (59), while OpiA was found to be a phosphatidylinositol 3-kinase delaying phagosomal maturation (17). It is possible that AnmK is a T6SS effector, which potentially competes with OpiA for secretion by T6SS. Another possibility is that either AnmK modulates OpiA expression levels or AnmK directly regulates OpiA function. Intriguingly, *anmK* is missing in F. tularensis subsp. *holarctica* and is expressed in two separate open reading frames in F. tularensis subsp. *tularensis* (15, 60). Surprisingly, the addition of *opiB_1_*_–_*_3_* to *anmK* and *opiA* background (Δ*pdpD* Δ*pdpC* mutant) reverts the intermediate phenotype to parental strain-like killing of G. mellonella larvae (Fig. 3C). This shows that further studies are necessary to fully understand the role of these proteins in infection.

Several different environmental signals, such as biotin, iron limitation, pH changes, oxidative stress, or starvation, were identified to increase FPI transcription or IglC production (50, 61–64). Nevertheless, our understanding of what triggers T6SS assembly remains limited. Here, we show that PEG, next to KCl and incubation on PBS agarose pads, activates T6SS assembly in F. tularensis subsp. *novicida* (Fig. 5A) (5, 10). It is still unclear which physiological signal is mimicked by incubation on PBS agarose pads and 10% PEG treatment. However, we show that orphan response regulator PmrA is dispensable for both of the two T6SS activation methods (Fig. 5D to F). The demonstration that PEG activates *Francisella* T6SS expands the toolbox for *Francisella* T6SS research, as it allows robust T6SS activation that is compatible with downstream analyses without exposing cells to high KCl concentration, which could potentially stress the cells.

In summary, we demonstrate that G. mellonella larvae are an easy to handle and robust *in vivo* infection model for studying *Francisella* virulence and its T6SS. Moreover, this model makes it possible to uncover new functions and interactions between T6SS components as shown for AnmK and OpiA. Further investigations about why some effectors are more toxic in one infection model than another will lead to a more detailed understanding of the mode of action of different effectors. Intriguingly, a well-characterized arthropod *in vivo* model might help to study *Francisella* traits important for persistence in the environment and in potential reservoir hosts.

## MATERIALS AND METHODS

### Bacterial strains.

F. tularensis subsp. *novicida* U112 and derivative strains were grown in brain heart infusion (BHI) broth with aeration or on BHI agar plates at 37°C. The medium was supplemented with 0.1% l-cysteine (Acros Organics) and 100 μg/ml ampicillin (AppliChem) for overnight cultures and plates. Escherichia coli DH5α λpir and derivative strains were aerobically grown in Luria broth (LB) or on agar plates supplemented with 50 μg/ml kanamycin at 37°C. All strains are listed in Table S1 in the supplemental material.

### Bacterial mutagenesis.

F. tularensis subsp. *novicida* in-frame deletion mutants were created with suicide vector pDMK3 (66) as reported previously (see Table S2 in the supplemental material) (5, 67). In brief, pDMK3 containing a DNA sequence of interest, including homology arms (750 bp each), was introduced into a donor E. coli strain from Harms and Dehio (68) and conjugated into F. tularensis subsp. *novicida*. For conjugation, liquid cultures of recipient F. tularensis subsp. *novicida* and donor E. coli strains were grown until an optical density at 600 nm (OD_600_) of 1 was reached. Day cultures were washed once in LB and 1 ml of both donor and recipient strain culture was concentrated and mixed together. Conjugation took place on an LB agar plate supplemented with 300 μM 2,6-diaminopimelic acid at 25°C overnight. Then, the mixture was transferred on Mueller-Hinton agar plates supplemented with 0.1% l-cysteine, 0.1% d-glucose (Millipore), 0.1% fetal calf serum (BioConcept), 100 μg/ml ampicillin, and 15 μg/ml kanamycin to select for recipients containing the suicide vector. After incubation at 37°C for 2 days, colonies were restreaked on BHI agar plates supplemented with 0.1% l-cysteine, 100 μg/ml ampicillin, and 15 μg/ml kanamycin. Negative selection was carried out on LB agar plates supplemented with 0.1% l-cysteine, 5% sucrose, and 100 μg/ml ampicillin, which were incubated at room temperature for several days. All plasmids and remaining peptides of in-frame deletions are listed in Table S2. All cloning products were sequenced, and sites of homologous recombination were verified by PCR.

### Galleria mellonella infections.

Weight and aged defined Galleria mellonella larvae from TruLarv (BioSystems Technology) were used for all infection experiments. For each experiment and condition, 10 randomly chosen larvae were infected as previously described (69). F. tularensis subsp. *novicida* strains were prepared as follows. Day cultures of bacterial strains from plates were inoculated at an OD_600_ of 0.2 and grown without antibiotics as described above for 3 h. Then, cultures were washed once with Dulbecco’s phosphate saline buffer without CaCl_2_ and MgCl_2_ (PBS; Sigma), and OD_600_ was adjusted to 1 in PBS. Ten-fold dilutions in PBS were carried out. Ten microliters of the 10^8^, 10^6^, or 10^4^ CFU/ml dilution (10^6^, 10^4^, or 10^2^ CFU per injection) was used for injection into the second left proleg with a Hamilton syringe (10-μl volume, 26s ga bevel tip, needle length of 51 mm; Sigma-Aldrich). All infected larvae per condition were placed in one petri dish (Greiner Bio-One) and incubated at 37°C for 5 days. Survival was scored manually every 24 h. Death was defined as no movement of legs, head, or body. Pupated larvae were considered alive as long as they exhibited any movement but were censored and not considered for calculating the percentage of surviving larvae. As control for proper handling, each experiment included larvae injected with PBS.

Petri dishes with 10 dead G. mellonella larvae and after 5 days all remaining G. mellonella larvae were incubated at −20°C overnight before disposal.

### Plating of inoculum.

The prepared 10-fold dilution series of F. tularensis subsp. *novicida* strains was also used to determine the actual inoculum concentration. A total of 100 μl of the calculated 10^3^ CFU/ml dilution was plated on Mueller-Hinton agar plates supplemented with 0.1% l-cysteine, 0.1% d-glucose (Millipore), 0.1% fetal calf serum (BioConcept), and 100 μg/ml ampicillin. The plates were incubated for 2 days at 37°C, and colonies were counted afterward.

### T6SS-dependent secretion assay.

Overnight cultures were washed twice with PBS and then resuspended in BHI and used for inoculation of day cultures without antibiotics at an OD_600_ of 0.2. After 3.5 h, the OD_600_ was adjusted to 2, and the bacterial cultures were washed twice with PBS and resuspended in 1 ml of BHI without l-cysteine. Then, 1 ml of 20% polyethylene glycol 4000 (Sigma-Aldrich) in BHI without l-cysteine was added so that a final PEG 4000 concentration of 10% was achieved. The cultures were incubated at 37°C shaking for 1 h. Afterward, 1 ml of the PEG 4000-treated samples was centrifuged at 16,000 × *g* for 1.5 min. A total of 0.9 ml of supernatant was used for trichloroacetic acid (TCA)/acetone protein precipitation. In brief, 100 μl of 100% TCA (wt/vol) (Sigma-Aldrich) was added to the harvested supernatants, followed by incubation at 4°C for 10 min with mixing in between. After centrifugation at 18,000 × *g* and 4°C for 5 min, the precipitated proteins were washed twice with cold acetone (Merk Millipore) and left to dry at room temperature. Then, the precipitated proteins were resuspended in 40 μl 1× lithium dodecyl sulfate (LDS) buffer (Thermo Fisher). The remaining bacterial cells were resuspended in 100 μl PBS, boiled at 95°C for 10 min, and sonicated afterwards. Thirty microliters of these samples were mixed with 10 μl 4× LDS buffer.

### SDS-PAGE and Western blotting.

Samples prepared for the T6SS-dependent secretion assay (see above) were supplemented with 4 μl of 1 M dithiothreitol (DTT; Roche) and incubated at 72°C for 10 min. Then, 20 μl of the samples was loaded on 10% polyacrylamide gels, and proteins were separated by gel electrophoresis. For immunodetection, proteins were transferred to a nitrocellulose membrane (25 V for 45 min). After blocking of the nitrocellulose membrane in 5% milk in Tris‐buffered saline containing Tween 0.1% (TBST) at room temperature for 2 h and three washing steps with TBST for 5 min each, the nitrocellulose membrane was incubated with the primary antibody at room temperature for 2 h. Primary α-IglC antibody (polyclonal antibody raised in rabbit; Genescript) was used at a final concentration of 1 μg/ml in 5% milk in TBST. Incubation for 1 h with secondary antibody α-rabbit conjugated to horseradish peroxidase (Jackson ImmunoResearch) at a final concentration of 30 ng/ml in 5% milk in TBST followed. LumiGLO chemiluminescent substrate (KPL) was used for detection of peroxidase on a gel imager (GE ImageQuant LAS 4000). Exposure time is given in the figure legend.

### Live-cell fluorescence imaging.

Microscope set up was described previously (5, 11, 70). The Nikon Ti-E inverted motorized microscope was equipped with a Perfect Focus system and a Plan Apo 1003 Oil Ph3 DM (NA 1.4) objective lens. Fluorescence was excited and filtrated with a SPECTRA X light engine (Lumencor) along with an ET-GFP (Chroma number 49002) filter set. The exposure time for each channel was set to 150 ms. Images were collected with a scientific complementary metal oxide semiconductor (sCMOS) camera pco.edge 4.2 with a pixel size of 65 nm (PCO) and VisiView software (Visitron). For imaging, day cultures of F. tularensis subsp. *novicida* parental and mutant strains were inoculated from plate at an OD_600_ of 0.2 without antibiotics. At an OD_600_ of 1, the cultures were concentrated in phosphate saline buffer to an OD_600_ of 10. A total of 1.5 μl of the concentrated cultures was then spotted on a pad consisting of 1% agarose in phosphate saline buffer. The agarose pad was covered with a cover slip and incubated at 37°C for 1 h before imaging at 30°C and 95% humidity (T-unit; Okolab). To monitor T6SS activation through PEG 4000 treatment (see T6SS-dependent secretion assay), 1.5 μl of liquid culture was spotted on a pad consisting of 1% agarose in BHI, covered by a coverslip, and imaged immediately. Images were collected every 30 s for 5 min.

### Image analysis.

Image analysis was carried out with Fiji software (71) as previously described (5, 70, 72). Images in the same subfigure were set to the same contrast values for comparison of fluorescent signal intensities. For quantification of T6SS assemblies per bacterium within 5 min, the “temporal color code” function was used together with the “Cell Counter” plugin.

### Statistical analysis.

Three infection experiments with independent G. mellonella larvae batches were performed. Mutants of a given set were tested in the same infection experiments. Pooled and single survival plots were calculated with Prism 8 (GraphPad Software). For more clarity, the graphs contain only data of the indicated mutant and the controls (G. mellonella larvae treated with PBS and infected with parental strain and T6SS) of the whole experiment. Thus, for a given set of mutants, the controls are the same for individual graphs. Standard errors were calculated for pooled survival plots. The log rank (Mantel-Cox) test in combination with Bonferroni corrected threshold (significance level, 0.05; number of comparisons, 6) was used to determine if compared curves are significantly different. *P* values are given in the figure legends.

Number of T6SS assemblies per bacterium was quantified in biological replicates. The smallest number of analyzed bacteria for a data set is given in the figure legend. Means with standard deviation were calculated. To test for significant differences in means, the Tukey’s multiple-comparison test with a confidence level of 95% in Prism 8 (GraphPad software) was used.
