# Supplementary material for: Type VI Secretion System and Its Effectors PdpC, PdpD, and OpiA Contribute to Francisella Virulence in Galleria mellonella Larvae
Source: Infect Immun. 2021 Jun 16;89(7):e00579-20. doi: 10.1128/IAI.00579-20 (PMC8208517; doi:10.1128/IAI.00579-20)
Supplement: Supplemental file 1 — Fig. S1 to S4; Movie S1 and S2 legends; Tables S1 and S2. Download IAI.00579-20-s0001.pdf, PDF file, 1.39 MB [file iai.00579-20-s0001.pdf]

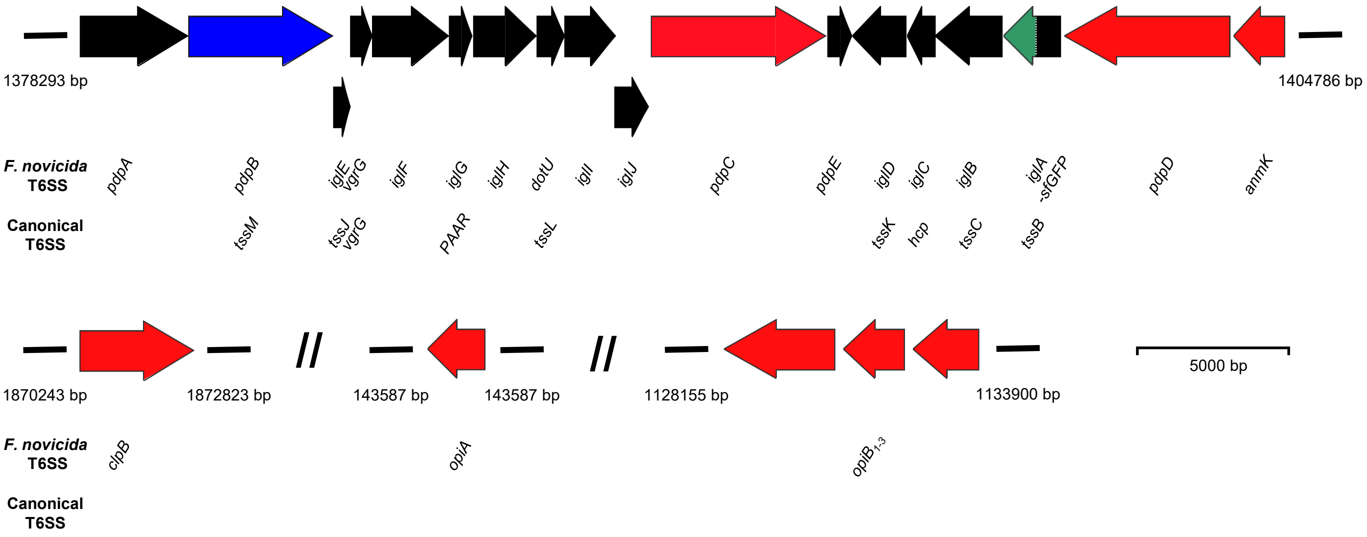

Figure S1: Scheme of *F. novicida* T6SS encoding genes (FPI, *clpB* and T6SS dependent secreted components *opiA* and *opiB*$_{1\text{-}3}$). Gene functions are assigned based on literature cited in the main text. *Francisella* and canonical T6SS nomenclature is shown. In black: genes that are not discussed in this study. In red: genes of interest, which were deleted in-frame deleted for this study. In blue: *pdpB*, in-frame deletion of this gene was used as T6SS negative control. Green represents fluorophore sfGFP, which was used to visualize sheath component *iglA*. Genes are drawn in scale.

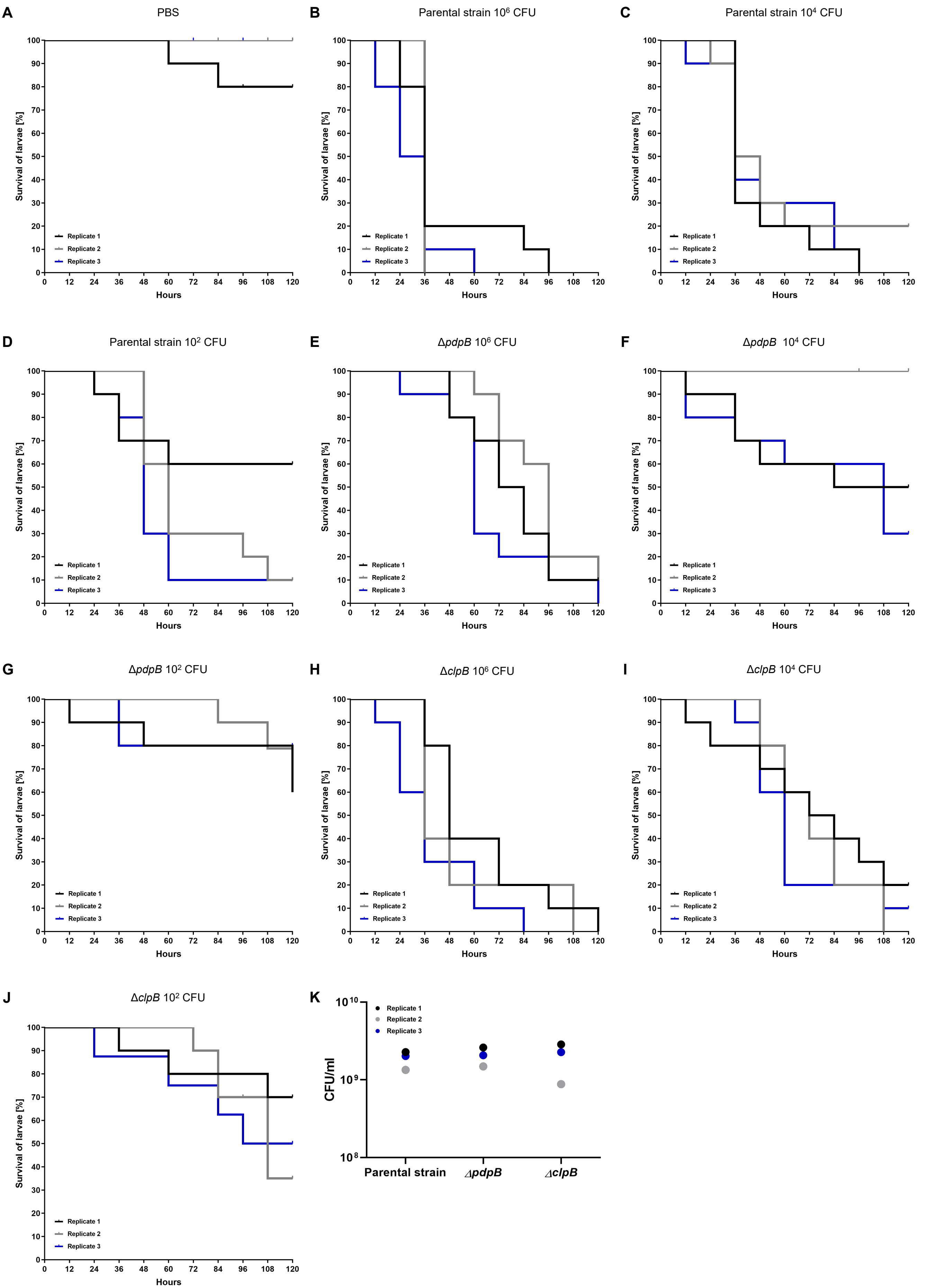

Figure S2: Individual survival curves for Figure 2. Individual survival curves of three individual experiments. Black: replicate 1, grey: replicate 2, blue: replicate 3. State of larvae was monitored every 12 h. Pupating larvae were censored (dashes). *G. mellonella* larvae (n_0=10) were treated with **A)** PBS and infected with **B-D)** *F. novicida* U112 *iglA-sfGFP* (**parental strain**), **E-G)** Δ*pdpB* (**T6SS negative control**) and **H-J)** Δ*clpB* with a calculated infection inocula of **B, E, H)** 10^6 CFU, **C, F, I)** 10^4 CFU and **D, G, J)** 10^2 CFU. **K)** CFU concentrations of the used infection inocula at OD_{600} of 1 for the individual experiments.

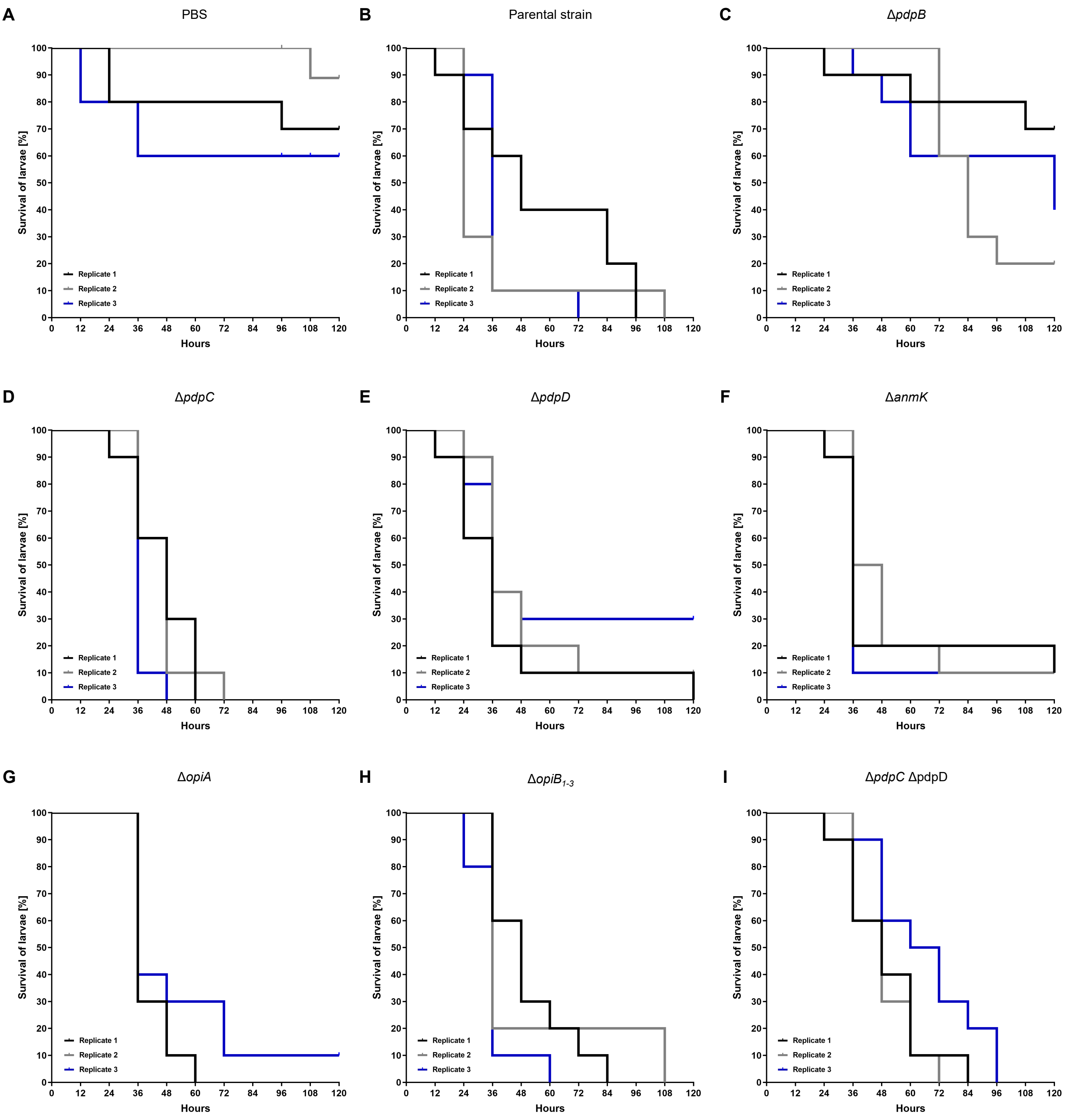

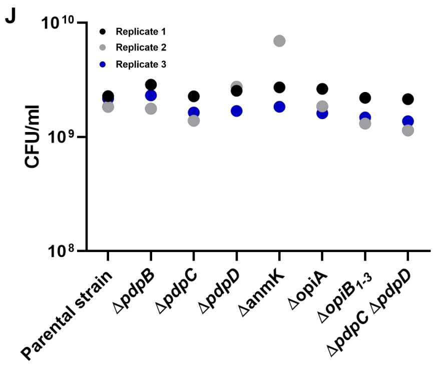

Figure S3: Individual survival curves for Figure 3. Individual survival curves of three individual experiments. Black: replicate 1, grey: replicate 2, blue: replicate 3. State of larvae was monitored every 12 h. Pupating larvae were censored (dashes). *G. mellonella* larvae (n_0=10) were treated with **A)** PBS and infected with **B)** *F. novicida* U112 *iglA-sfGFP* (**parental strain**), **C)** Δ*pdpB* (**T6SS negative control**), **D)** Δ*pdpC*, **E)** Δ*pdpD*, **F)** Δ*anmK*, **G)** Δ*opiA*, **H)** Δ*opiB*$_{1\text{-}3}$ and **I)** Δ*pdpC* Δ*pdpD*. **J)** CFU concentrations of the used infection inocula at OD_{600} of 1 for the individual experiments.

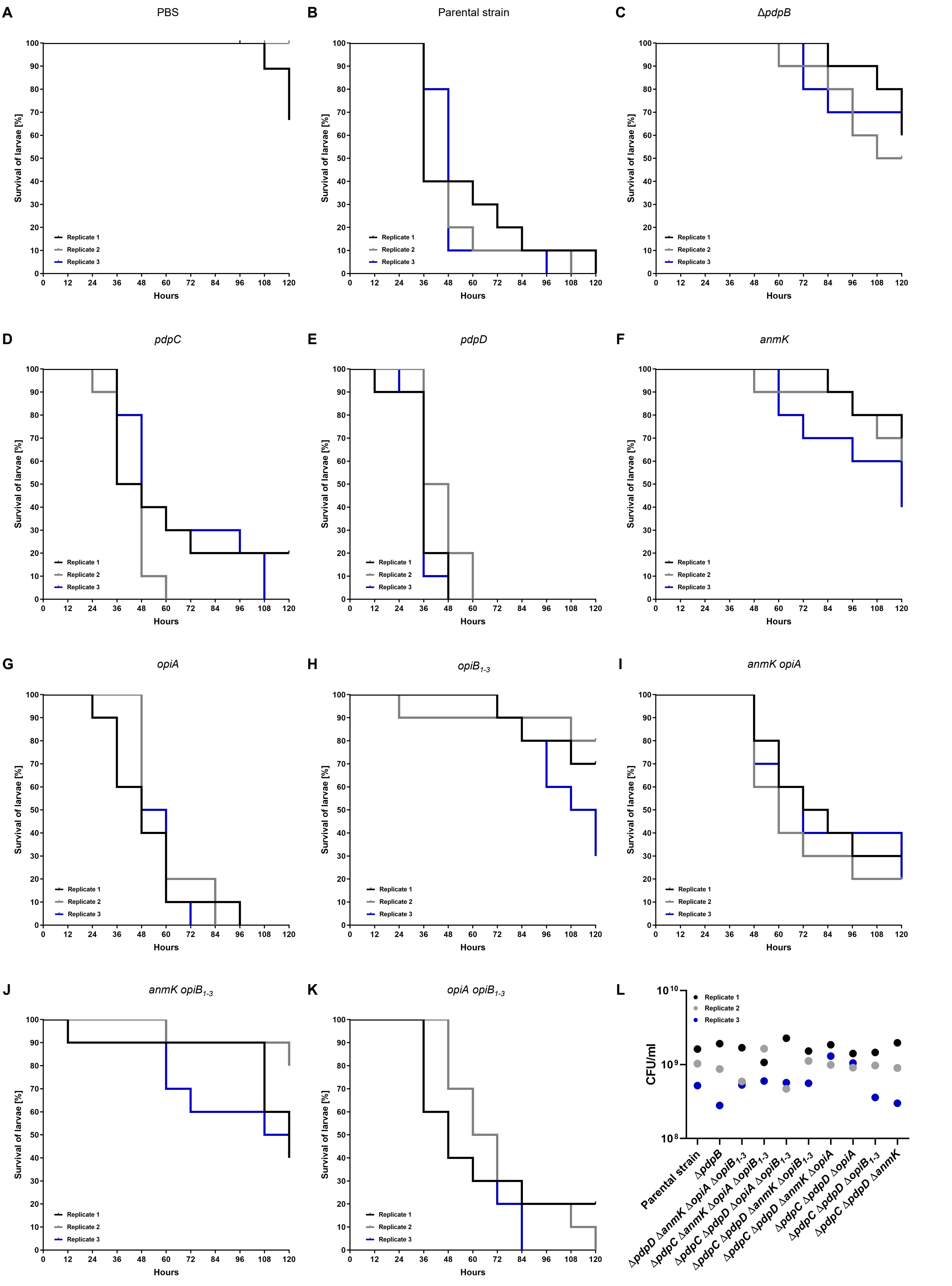

Figure S4: Individual survival curves for Figure 4. Individual survival curves of three individual experiments. Black: replicate 1, grey: replicate 2, blue: replicate 3. State of larvae was monitored every 12 h. Pupating larvae were censored (dashes). *G. mellonella* larvae (n_0=10) were treated with **A)** PBS and infected with **B)** *F. novicida* U112 *iglA-sfGFP* (**parental strain**), **C)** Δ*pdpB* (**T6SS negative control**), **D)** Δ*pdpD* Δ*anmK* Δ*opiA* Δ*opiB*$_{1\text{-}3}$ (***pdpC***), **E)** Δ*pdpC* Δ*anmK* Δ*opiA* Δ*opiB*$_{1\text{-}3}$ (***pdpD***), **F)** Δ*pdpC* Δ*pdpD* Δ*opiA* Δ*opiB*$_{1\text{-}3}$ (***anmK***), **G)** Δ*pdpC* Δ*pdpD* Δ*anmK* Δ*opiB*$_{1\text{-}3}$ (***opiA***), **H)** Δ*pdpC* Δ*pdpD* Δ*anmK* Δ*opiA* (***opiB***$_{1\text{-}3}$), **I)** Δ*pdpC* Δ*pdpD* Δ*opiB*$_{1\text{-}3}$ (***anmK opiA***), **J)** Δ*pdpC* Δ*pdpD* Δ*opiA* (***anmK opiB***$_{1\text{-}3}$) and **K)** Δ*pdpC* Δ*pdpD* Δ*anmK* (***opiA opiB***$_{1\text{-}3}$). **L)** CFU concentrations of the used infection inocula at OD_{600} of 1 for the individual experiments.

Supplemental movies

- **Movie S1: Functional T6SS dynamics in parental strain and mutants.** T6SS dynamics (IglA-sfGFP) was monitored in *F. novicida* U112 *iglA-sfGFP* (**parental strain**), Δ*pdpB* (**T6SS negative control**), Δ*clpB*, Δ*pdpC*, Δ*pdpD*, Δ*anmK*, Δ*opiA*, Δ$opiB_{1\text{-}3}$, Δ*pdpC* Δ*pdpD*, Δ*pdpD* Δ*anmK* Δ*opiA* Δ$opiB_{1\text{-}3}$ (***pdpC***), Δ*pdpC* Δ*anmK* Δ*opiA* Δ$opiB_{1\text{-}3}$ (***pdpD***), Δ*pdpC* Δ*pdpD* Δ*opiA* Δ$opiB_{1\text{-}3}$ (***anmK***), Δ*pdpC* Δ*pdpD* Δ*anmK* Δ$opiB_{1\text{-}3}$ (***opiA***), Δ*pdpC* Δ*pdpD* Δ*anmK* Δ*opiA* (***$opiB_{1\text{-}3}$***), Δ*pdpC* Δ*pdpD* Δ$opiB_{1\text{-}3}$ (***anmK opiA***), Δ*pdpC* Δ*pdpD* Δ*opiA* (***anmK $opiB_{1\text{-}3}$***) and Δ*pdpC* Δ*pdpD* Δ*anmK* (***opiA $opiB_{1\text{-}3}$***) for 5 min at a frame rate of 2 frames per minute. Two representative time-lapse image series (merge of phase contrast and GFP channel) for each strain are shown. Fields of view are 39 x 26 µm. Scale bars represent 5 µm. Videos play at a frame rate of 5 frames per second.

- **Movie S2: Detailed examples of individual T6SS assemblies in parental strain and mutants.** T6SS dynamics (IglA-sfGFP) was monitored in *F. novicida* U112 *iglA-sfGFP* (**parental strain**), Δ*pdpB* (**T6SS negative control**), Δ*clpB*, Δ*pdpC*, Δ*pdpD*, Δ*anmK*, Δ*opiA*, Δ$opiB_{1\text{-}3}$, Δ*pdpC* Δ*pdpD*, Δ*pdpD* Δ*anmK* Δ*opiA* Δ$opiB_{1\text{-}3}$ (***pdpC***), Δ*pdpC* Δ*anmK* Δ*opiA* Δ$opiB_{1\text{-}3}$ (***pdpD***), Δ*pdpC* Δ*pdpD* Δ*opiA* Δ$opiB_{1\text{-}3}$ (***anmK***), Δ*pdpC* Δ*pdpD* Δ*anmK* Δ$opiB_{1\text{-}3}$ (***opiA***), Δ*pdpC* Δ*pdpD* Δ*anmK* Δ*opiA* (***$opiB_{1\text{-}3}$***), Δ*pdpC* Δ*pdpD* Δ$opiB_{1\text{-}3}$ (***anmK opiA***), Δ*pdpC* Δ*pdpD* Δ*opiA* (***anmK $opiB_{1\text{-}3}$***) and Δ*pdpC* Δ*pdpD* Δ*anmK* (***opiA $opiB_{1\text{-}3}$***) for 5 min at a frame rate of 2 frames per minute. Two representative time-lapse image series (merge of phase contrast and GFP channel) for each strain are shown. Fields of view are 3.3 x 3.3 µm. Scale bars represent 1 µm. Videos play at a frame rate of 5 frames per second.

Table S1: Strains used in this study, related to Material and methods

Organism	Genotype	Plasmid	Relevant features	Source
Francisella novicida U112	*iglA-sfGFP*		C-terminal chromosomal fusion of *sfGFP* to *iglA* (parental strain)	(1)
	iglA-sfGFP Δ*pdpB*		In-frame deletion of *pdpB* (T6SS negative control)	(2)
	iglA-sfGFP Δ*iglC*		In-frame deletion of *iglC* (negative control for α-IglC antibody)	(3)
	iglA-sfGFP Δ*clpB*		In-frame deletion of *clpB*	(2)
	iglA-sfGFP Δ*pdpC*		In-frame deletion of *pdpC*	(2)
	iglA-sfGFP Δ*pdpD*		In-frame deletion of *pdpD*	(2)
	iglA-sfGFP Δ*anmK*		In-frame deletion of *anmK*	(2)
	iglA-sfGFP Δ*opiA*		In-frame deletion of *opiA*	This study
	iglA-sfGFP Δ$opiB_{1\text{-}3}$		In-frame deletion of $opiB_1$,$opiB_2$ and $opiB_3$	This study
	iglA-sfGFP Δ*pdpC* Δ*pdpD*		In-frame deletion of *pdpC* and *pdpD*	(2)
	iglA-sfGFP Δ*pdpD* Δ*anmK* Δ*opiA* Δ$opiB_{1\text{-}3}$		In-frame deletion of *pdpD*, *anmK*, *opiA* and $opiB_{1\text{-}3}$ (*pdpC*)	This study
	iglA-sfGFP Δ*pdpC* Δ*anmK* Δ*opiA* Δ$opiB_{1\text{-}3}$		In-frame deletion of *pdpC*, *anmK*, *opiA* and $opiB_{1\text{-}3}$ (*pdpD*)	This study
	iglA-sfGFP Δ*pdpC* Δ*pdpD* Δ*opiA* Δ$opiB_{1\text{-}3}$		In-frame deletion of *pdpC*, *pdpD*, *opiA* and $opiB_{1\text{-}3}$ (*anmK*)	This study
	iglA-sfGFP Δ*pdpC* Δ*pdpD* Δ*anmK* Δ$opiB_{1\text{-}3}$		In-frame deletion of *pdpC*, *pdpD*, *anmK* and $opiB_{1\text{-}3}$ (*opiA*)	This study
	iglA-sfGFP Δ*pdpC* Δ*pdpD* Δ*anmK* Δ*opiA*		In-frame deletion of *pdpC*, *pdpD*, *anmK* and *opiA* ($opiB_{1\text{-}3}$)	This study
	iglA-sfGFP Δ*pdpC* Δ*pdpD* Δ$opiB_{1\text{-}3}$		In-frame deletion of *pdpC*, *pdpD* and $opiB_{1\text{-}3}$ (*anmK opiA*)	This study
	iglA-sfGFP Δ*pdpC* Δ*pdpD* Δ*opiA*		In-frame deletion of *pdpC*, *pdpD* and *opiA* (*anmK* $opiB_{1\text{-}3}$)	This study
	iglA-sfGFP Δ*pdpC* Δ*pdpD* Δ*anmk*		In-frame deletion of *pdpC*, *pdpD* and *anmK* (*opiA* $opiB_{1\text{-}3}$)	(2)
	iglA-sfGFP Δ*pmrA*		In-frame deletion of *pmrA*	This study

Table S2: Plasmids and primers used to generate mutants, related to Material and Methods.

Plasmid Name	Peptide scar [amino acids]	Primers	Sequence 5’-3’ [base pairs]
pDMK3 *ΔopiA*	MKNFEVIRKDFFSHLCNLLN*	dFTN_0131_Xho1_1.FOR	TCAGTACTCGAGAGTTTATTTTTAATCCACATAAGC
		dFTN_0131_1.REV	TACGCAAAGATTTTTCTCATTTGTGTAATTTGTTG
		dFTN_0131_2.FOR	AAATGAGAAAAATCTTTGCGTATTACTTC
		dFTN_0131_Not1_2.REV	TCAGTAGCGGCCGCGTCAACCATATAACAAAGGC
		dFTN_0131_Det.FOR	TCCGGAAAATATCGTTGGAGT
		dFTN_0131_Det.REV	TGGCAGTCTTTAGAGGAGCT
pDMK3 *ΔopiB*$_{1\text{-}3}$	MAIDLLKLQKSNGLPGFLL*	dFTN_1069-71_Xho1_1.FOR	TCAGTACTCGAGATAATTTATAGTCCTAGTAAAGTTACT
		dFTN_1069-71_1.REV	AAAACTACAAAAAAGTAATGGATTACCAGGA
		dFTN_1069-71_2.FOR	GTAATCCATTACTTTTTTGTAGTTTTAATAGATCAATAGC
		dFTN_1069-71_Not1_2.REV	TCAGTAGCGGCCGCAATATCATCTTGAGTTATCGG
		dFTN_1069-71_Det.FOR	TTCGTGATCAGCTACAAGGC
		dFTN_1069-71_Det1.REV	AGCTTTGTAAACCTCCAAGTTCT
pDMK3 *ΔpmrA*	MRILLAEDDLFVQKDKVIK*	dFTN_1465_1_Xho1.FOR	TCAGTACTCGAGATATTTTACCCCTTTTGACTG
		dFTN_1465_1.REV	CTTTTGTACAAAAAGATCATCTTCAGCCA
		dFTN_1465_2.FOR	GAAGATGATCTTTTTGTACAAAAGGATAAAGTAATTAAG
		dFTN_1465_2_Not1.REV	TCAGTAGCGGCCGCATGACAAAGTATTGACCTGC
		dFTN_1465_Det.FOR	GGGCGATTGTAGCAAGAAAG
		dFTN_1465_Det.REV	CATCCCAGCGAACCTTTTTA

References

1. Clemens DL, Ge P, Lee B-Y, Horwitz MA, Zhou ZH. 2015. Atomic Structure of T6SS Reveals Interlaced Array Essential to Function. Cell 160:940–951.

2. Brodmann M, Dreier RF, Broz P, Basler M. 2017. Francisella requires dynamic type VI secretion system and ClpB to deliver effectors for phagosomal escape. Nat Commun 8:15853.

3. Brodmann M, Heilig R, Broz P, Basler M. 2018. Mobilizable Plasmids for Tunable Gene Expression in Francisella novicida. Front Cell Infect Microbiol 8.
